# A novel hand-assisted laparoscopic versus conventional laparoscopic right hemicolectomy for right colon cancer: study protocol for a randomized controlled trial

**DOI:** 10.1186/s13063-017-2084-3

**Published:** 2017-07-26

**Authors:** Xuyang Yang, Qingbin Wu, Chengwu Jin, Wanbin He, Meng Wang, Tinghan Yang, Mingtian Wei, Xiangbing Deng, Wenjian Meng, Ziqiang Wang

**Affiliations:** 1 Department of Gastrointestinal Surgery, West China Hospital, Sichuan University, No. 37 Guo Xue Alley, Chengdu, 610041 Sichuan Province China; 2 Department of Gastrointestinal Surgery, The Fifth People’s Hospital of Chengdu, Chengdu, 611130 Sichuan Province China

**Keywords:** Hand-assisted laparoscopic surgery, Complete mesocolic excision, D3 lymphadenectomy, No-touch isolation technique, Polymerase chain reaction, Non-inferiority, Randomized controlled trial

## Abstract

**Background:**

Although conventional laparoscopic and hand-assisted laparoscopic surgery for colorectal cancer is widely used today, there remain many technical challenges especially for right colon cancer in obese patients. Herein, we develop a novel hand-assisted laparoscopic surgery (HALS) with complete mesocolic excision (CME), D3 lymphadenectomy, and a total “no-touch” isolation technique (HALS-CME) in right hemicolectomy to overcome these issues. According to previous clinic practice, this novel procedure is not only feasible and safe but has several technical merits. However, the feasibility, short-term minimally invasive virtues, long-term oncological superiority, and potential total “no-touch” isolation technique benefits of HALS-CME should be confirmed by a prospective randomized controlled trial.

**Methods/design:**

This is a single-center, open-label, noninferiority, randomized controlled trial. Eligible participants will be randomly assigned to the HALS-CME group or to the laparoscopic surgery with CME, D3 lymphadenectomy, and total “no-touch” isolation technique (LAP-CME) group, or to conventional laparoscopic surgery with CME and D3 lymphadenectomy (cLAP) group at a 1:1:1 ratio using a centralized randomization list. Primary endpoints include safety, efficacy, and being oncologically clear, and 3-year disease-free, progression-free, and overall survival. Second endpoints include operative outcomes (operation time, blood loss, and incision length), pathologic evaluation (grading the plane of surgery, length of proximal and distal resection margins, distance between the tumor and the central arterial high tie, distance between the nearest bowel wall and the same high tie, area of mesentery resected, width of the chain of lymph-adipose tissue, length of the central lymph-adipose chain, number of harvested lymph nodes), and postoperative outcomes (pain intensity, postoperative inflammatory and immune responses, postoperative recovery).

**Discussion:**

This trial will provide valuable clinical evidence for the feasibility, safety, and potential total “no-touch” isolation technique benefits of HALS-CME for right hemicolectomy. The hypothesis is that HALS-CME is feasible for the radical D3 resection of right colon cancer and offers short-term safety and long-term oncological superiority compared with conventional laparoscopic surgery.

**Trial registration:**

ClinicalTrials.gov, NCT02625272. Registered on 8 December 2015.

**Electronic supplementary material:**

The online version of this article (doi:10.1186/s13063-017-2084-3) contains supplementary material, which is available to authorized users.

## Background

### Rationale

Since the first laparoscopic colon resection was performed in the 1990s, laparoscopic surgery had gained popularity for colon cancer, having fewer complications, a shorter length of hospital stay, and a quicker return to daily living and work when compared with traditional surgery [1, 2]. Furthermore, several randomized trials have demonstrated more favorable long-term cancer-specific or overall survival in laparoscopic colon surgery [3–5]. In recent years, progress in the treatment of colon cancer mainly includes that: 1) both European complete mesocolic excision (CME) with central vascular ligation (CVL) and Japanese D3 lymphadenectomy were demonstrated to have oncological superiority compared with traditional surgery [6–8]; 2) the feasibility and safety of laparoscopic right hemicolectomy with CME or D3 lymphadenectomy for right colon cancer has been confirmed [9–11].

Although it has a number of advantages, some drawbacks such as loss of tactile feedback, impaired hand-eye coordination, long operation time, and long learning curve still exist in laparoscopic surgery [12]. Moreover, these difficulties will become more obvious in laparoscopic right hemicolectomy for the high vascular variation in the right colon. In order to solve these problems, hand-assisted laparoscopic surgery (HALS) has been developed. Although HALS returns the sense of touch to the surgeon and the hand-assisted devices lower the threshold for surgeons to attempt laparoscopic techniques, people argue that HALS associated with rough visceral manipulation and the inadequate “no-touch” technique results in tumor cell spread [12, 13]. In addition, as far as we know, HALS with CME in right hemicolectomy is rarely reported in the literature.

Based on the above considerations, we developed a novel HALS with CME, D3 lymphadenectomy, and total “no-touch” isolation technique (HALS-CME) in right hemicolectomy to overcome these issues. According to our previous experience, this novel approach is not only feasible and safe but promises several technical merits.

### Objective and hypothesis

The objective of this trial is to evaluate the feasibility, short-term safety, long-term oncological superiority, and potential benefits of the “no-touch” isolation technique. The hypothesis is that the novel HALS-CME techniques is more consistent with the “no-touch” isolation technique, and is feasible for radical D3 lymphadenectomy, and could offer short-term safety, fast convalescence, and long-term oncological superiority.

## Methods and design

### Study design and setting

**Fig. 1:**
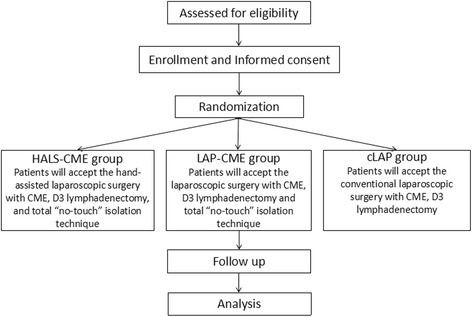
Participants flow through the trial. *cLAP* conventional laparoscopic surgery with CME and D3 lymphadenectomy, *CME* complete mesocolic excision, *HALS-CME* hand-assisted laparoscopic surgery with CME, D3 lymphadenectomy, and total “no-touch” isolation, *LAP-CME* laparoscopic surgery with CME, D3 lymphadenectomy, and total “no-touch” isolation

This is a single-center, open-label, noninferiority, randomized controlled trial undertaken at West China Hospital of Sichuan University (Chengdu, Sichuan, China), which performs 2000 colorectal cancer resections per year. The chief surgeon performs more than 400 laparoscopic-colorectal cancer resections per year. Participants will be randomly assigned to three parallel groups at a 1:1:1 ratio using a centralized randomization list. Patients assigned to the HALS-CME group will receive the hand-assisted laparoscopic surgery with CME, D3 lymphadenectomy, and total “no-touch” isolation technique (HALS-CME). Patients in the LAP-CME group will receive laparoscopic surgery with CME, D3 lymphadenectomy, and total “no-touch” isolation technique. Meanwhile, patients in cLAP group will receive conventional laparoscopic surgery with CME and D3 lymphadenectomy (Fig. 1). Blood samples and peritoneal washes will be collected from each participant during the operative procedure. Recruitment began on December 2015, and the trial is expected to proceed for 3 years.

### Ethical considerations and registration

This trial protocol has been approved by the Biological and Medical Ethics Committee of West China Hospital (2015 trial number (130)) and has been registered at the Clinical Trial Registry as NCT02625272 on 8 December 2015. All of the eligible participants will be informed of the potential risks and benefits of intervention in each group. A signed written informed consent will be obtained from each patient. All the results will be presented with the CONSORT (Consolidated Standards of Reporting Trials) statement (Additional file 1).

## Participants

Patients aged 18 to 80 years diagnosed with right colon cancer via abdominal enhanced computed tomography (CT) and total colonoscopy will be further screened for inclusion and exclusion.

### Inclusion criteria

Prior to enrollment in the study, patients must fulfill all of the following criteria:

1. Diagnosed with right colon cancer: ileocecum cancer, ascending colon cancer, hepatic flexure colon cancer
2. According to the 7th Edition of the American Joint Committee on Cancer (AJCC) Cancer Staging Manual, preoperative clinic stages: cT1-4a, N0-2a, M0
3. No preoperative radiochemotherapy for cancer
4. No past significant operative history
5. Eastern Cooperative Oncology Group (ECOG) performance status: 0 to 2
6. American Society of Anesthesiologists (ASA) classification: 1 to 3

### Exclusion criteria

Patients will be excluded for any of the following reasons:

1. Other sites of cancer such as transverse colon, descending colon, sigmoid colon, and rectum
2. Distant metastases evident on chest, abdominal, and pelvic CT scan
3. Emergency condition caused by cancer: bleeding, perforation, obstruction
4. Previous significant abdominal surgery (except appendectomy, cholecystotomy)
5. Severe mental disease
6. Malignant disease within the previous 5 years

## Outcome measures

### Primary outcomes

The primary endpoints are safety, efficacy, and being oncologically clear. Perioperative morbidity will be divided into intraoperative morbidity observed during the operation and postoperative morbidity observed during the hospital stay and within 30 days at the outpatient clinic. Postoperative morbidity will be assessed according to the Clavien-Dindo classification. Real-time polymerase chain reaction (RT-PCR) will be used to test whether the novel total “no-touch” isolation technique could potentially reduce tumor cells shedding into the portal circulation, peripheral circulation and peritoneal cavity. Other primary outcomes include 3-year disease-free survival (DFS), progression-free survival (PFS), overall survival (OS), and recurrence pattern (local recurrence, metastasis).

### Secondary outcomes

Secondary endpoints will be measured and recorded prospectively on a case report form (CRF). Intraoperative outcomes include operation time, blood loss, intraoperative complications, and incision length. Pathologic evaluation includes tumor size, grading the plane of surgery (mesocolic plane, intramesocolic plane, muscularis propria plane), length of proximal and distal resection margins, distance between the tumor and the central arterial high tie, distance between the nearest bowel wall and the same high tie, area of mesentery resected, width of the chain of lymph-adipose tissue (the width of the central lymph node region through the high tie), length of the central lymph-adipose chain (the length of the central lymph node region through the high tie), and number of harvested lymph nodes. Postoperative inflammation and immune response (procalcitonin (PCT), C-reactive protein (CRP), interleukin (IL)-6) will be measured at 24 h, 72 h, and 120 h postoperatively. A horizontal visual analogue scale with a 0 to 10 cm scale will be used to measure pain intensity on postoperative days 1, 3, and 5. Postoperative recovery including time to first flatus, liquid diet, and duration of hospital stay will be recorded daily in hospital.

## Follow-up

The follow-up is consistent with the National Comprehensive Cancer Network (NCCN) guidelines. Within 1 month after surgery, all the consecutive patients in this prospective study will meet the chief surgeon in the outpatient clinic for checking of wounds, recording any complications arising after discharge, and determining the optimal schedule of adjuvant chemotherapy according to the pathohistology. Then 3 months after surgery, and every 3 months for the first 2 years, participants will be followed-up. After 2 years, the surveillance interval will be every 6 months until 3 years or dropout. Data are collected prospectively, including physical examination, blood tests (blood cell count and blood chemistry), tumor markers (carcinoembryonic antigen (CEA), carbohydrate antigen 19-9 (CA 19-9)) every 3 months and CT scans of the chest and abdomen every 6 months. Colonoscopy will be performed 1 year after surgery and will be repeated at 3 years if no lesions are found.

## Safety and adverse events monitoring

An independent data and safety monitoring board organized by oncologists, gastrointestinal surgeons, and statisticians is responsible for overseeing the progress and safety of the study, including adverse events, morbidity, and drop-outs. All adverse events will be evaluated for severity. Any serious adverse events such as death, disability, and prolonged hospitalization will be recorded on the CRF and reported to the board and the Biological and Medical Ethics Committee of West China Hospital within 24 h. If the number of serious adverse events related to treatment is more than in our own database or as reported by other authors, patient enrollment will be terminated immediately and the Medical Ethics Committee will reassess the safety of the trial. The schedule of study visits and follow-up is shown in Table 1.

**Table 1:** Perioperative outcome parameters and schedule of study visits and follow-up

Measures	Preoperative	Intraoperative	Daily in-hospital study visit					Follow-up												
			POD 1	POD 2	POD 3	POD 4	POD 5	M1	M3	M6	M9	M12	M15	M18	M21	M24	M27	M30	M33	M36
Minimally invasive evaluation		X																		
Quality of specimens		X																		
VAS score			X		X		X													
Inflammatory parameters	X		X		X		X													
Short-term recovery			X	X	X	X	X													
Physcial examination	X							X	X	X	X	X	X	X	X	X		X		X
Tumor marker	X							X	X	X	X	X	X	X	X	X		X		X
Abdominal and pelvic CT scan	X									X		X		X		X		X		X
Colonoscopy	X											X				X				X
Adverse event		X	X	X	X	X	X	X	X	X	X	X	X	X	X	X	X	X	X	X

Preoperative study visits will be scheduled within 7 days before surgery

Minimally invasive evaluation includes operation time, blood loss, intraoperative complication, and incision length

Inflammatory parameters including procalcitonin (PCT), C-reactive protein (CRP), and interleukin (IL)-6 will be measured at 24 h, 72 h, and 120 h postoperatively

Short-term recovery including time to first flatus, liquid diet, and duration of hospital stay will be recorded daily in-hospital

Colonoscopy will be performed at 1 year after surgery and will be repeated at 3 years if no lesions are found

Tumor marker includes carcinoembryonic antigen (CEA) and carbohydrate antigen 19-9 (CA 19-9)

*CT* computed tomography, *M* Month, *POD* postoperative day, *VAS* visual analogue scale

## Sample size and statistical analysis

As the primary aim of the prospective trial is exploratory, no sample size calculation is performed. The statistical tests will be performed in the SPSS software program (version 13.0, SPSS, Chicago, IL, USA). A two-sided *P* value less than 0.05 will be considered statistically significant. Descriptive statistics will be used for baseline demographic and clinical characteristics of participants (i.e., mean and standard deviation for continuous variables, proportions for categorical variables). A χ^2^ test or Fisher’s exact test will be applied for categorical variables. Student’s *t* test or the Mann-Whitney *U* test will be used for continuous variables. Kaplan-Meier curves and log-rank analysis will be used to compare the differences in DFS and OS among the three parallel groups.

## Interventions

An experienced colorectal surgeon with more than 400 successful laparoscopic colorectal resections every year will perform all procedures in this trial.

### Experiment intervention

**Fig. 2:**
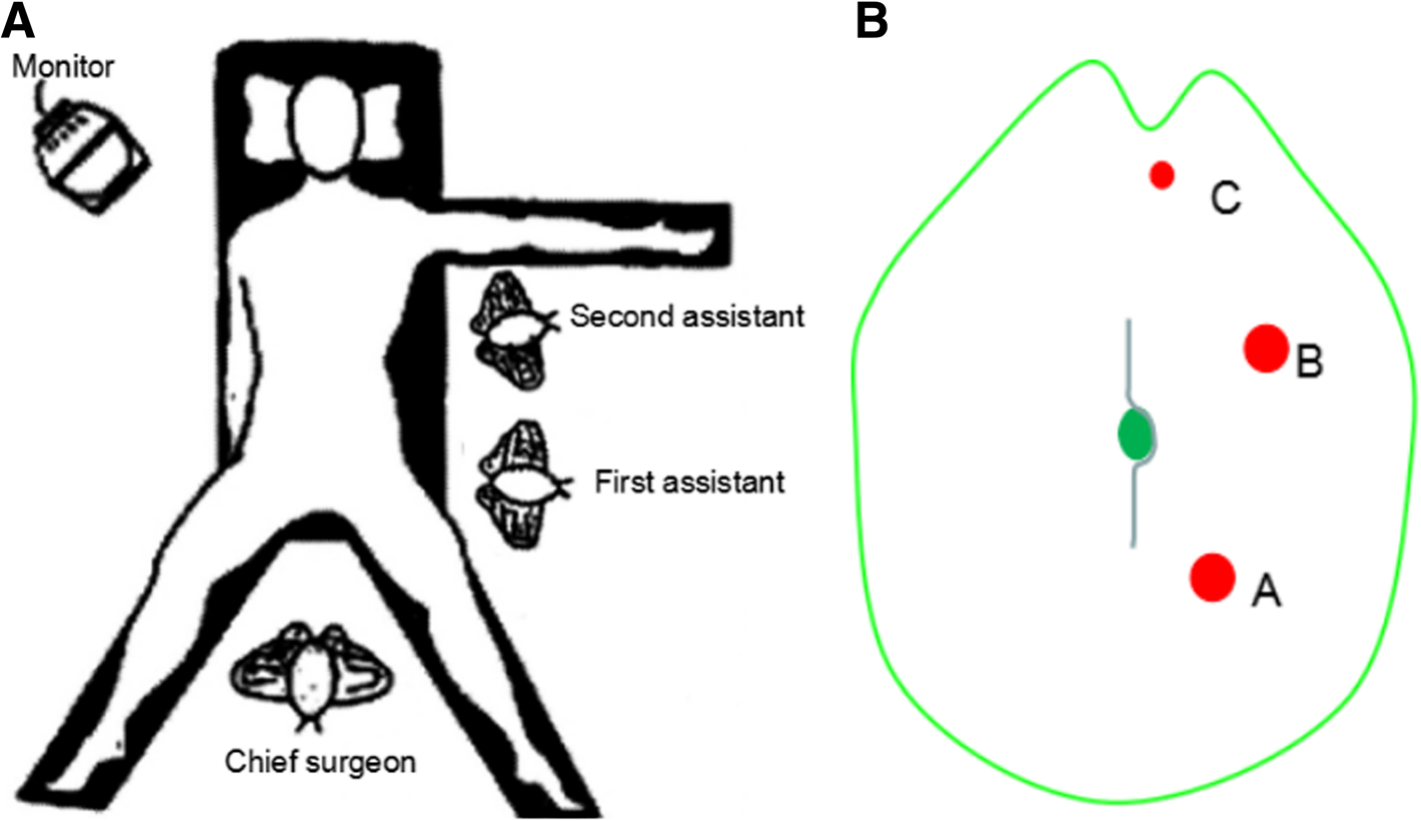
Operative position and trocar positions for the experimental intervention group. **a** The operative position for HALS-CME. **b** The trocar placement for HALS-CME. Trocars A and B = 12 mm, trocar C = 5 mm

After general anesthesia, the patient will be placed in the supine position with legs apart. The chief surgeon stands between the legs, and two assistants stand on the left side of the patient. The monitor is placed at the patient’s head. The positions of the chief surgeon, assistants and monitor are shown in Fig. 2a.

An initial 7-cm midline incision around the umbilicus will be made as the hand-assisted port and the Dextrus hand access device (Ethicon, USA) will be inserted through the umbilical incision. A 12-mm port (trocar A) in the left lower quadrant will be established as the surgeon’s dominant operation channel. In the upper left quadrant, one 12-mm port (trocar B) will be established to maintain a pneumoperitoneum of 12–15 mmHg, and the camera will be introduced through this port. Another 5-mm port (trocar C) slightly below the xiphoid will also be established as the assistant’s operation channel to retract the mesocolon and the stomach. The midline incision and trocars are shown in Fig. 2b.

**Fig. 3:**
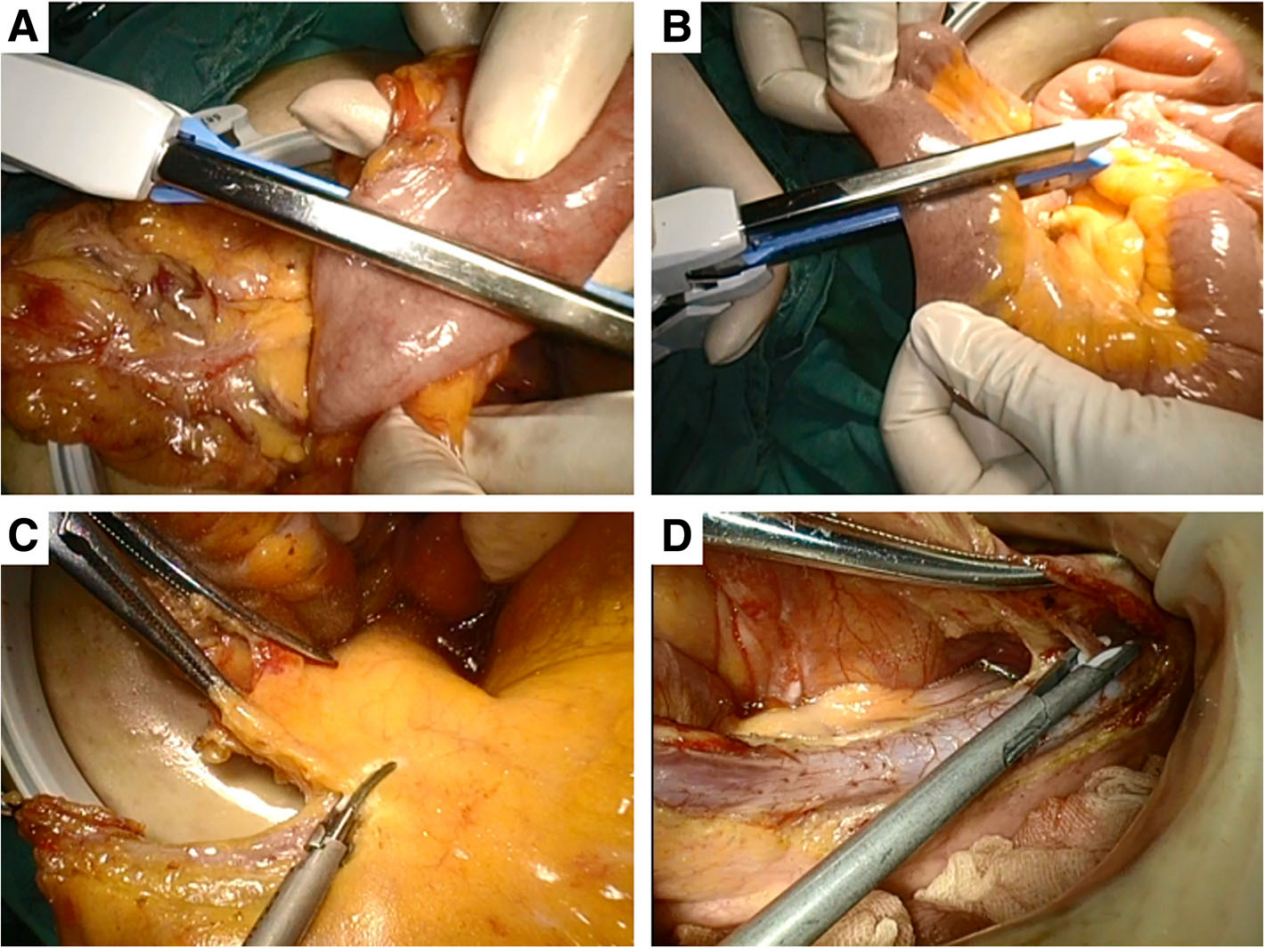
The extracorporeal surgical procedure for the HALS-CME group. **a** The transverse colon is divided after the right branch of the middle colic artery ligation. **b** The distal ileum is divided. **c** The terminal of the SMV is identified. **d** Dissection along the left side of the SMV proceeds up towards the origin of ileocolic pedicle, and the pedicle is divided

With the space created by the Dextrus, the whole operation procedure will be divided into extracorporeal and intracorporeal stages. Under direct visualization, the greater omentum, transverse colon, and terminal ileum can be exposed extracorporeally through the hand-assisted port. First, the transverse colon will be dragged out and the right branch of the middle colic vessels will be divided; after that, the transverse colon will also be divided to the left of the main trunk of the middle colic vessels and returned to the abdominal cavity (Fig. 3a). Second, the distal ileum from 6 to 10 cm proximal to the ileocecal valve as the proximal margin of resection is divided (Fig. 3b). The terminal of the superior mesenteric vein (SMV) can then be easily identified by holding and stretching the stump of the distal superior mesenteric vessels (Fig. 3c). We generally create a window around the terminal SMV, and dissection along the left side of the SMV will proceed up towards the origin of the ileocolic pedicle, and the pedicle will be divided extracorporeally (Fig. 3d). The small bowel and mesentery will then be returned to abdominal cavity. The chief surgeon’s left hand is put into the abdominal cavity through the hand-assisted port which facilitates maintaining a pneumoperitoneum of 12–15 mmHg.

**Fig. 4:**
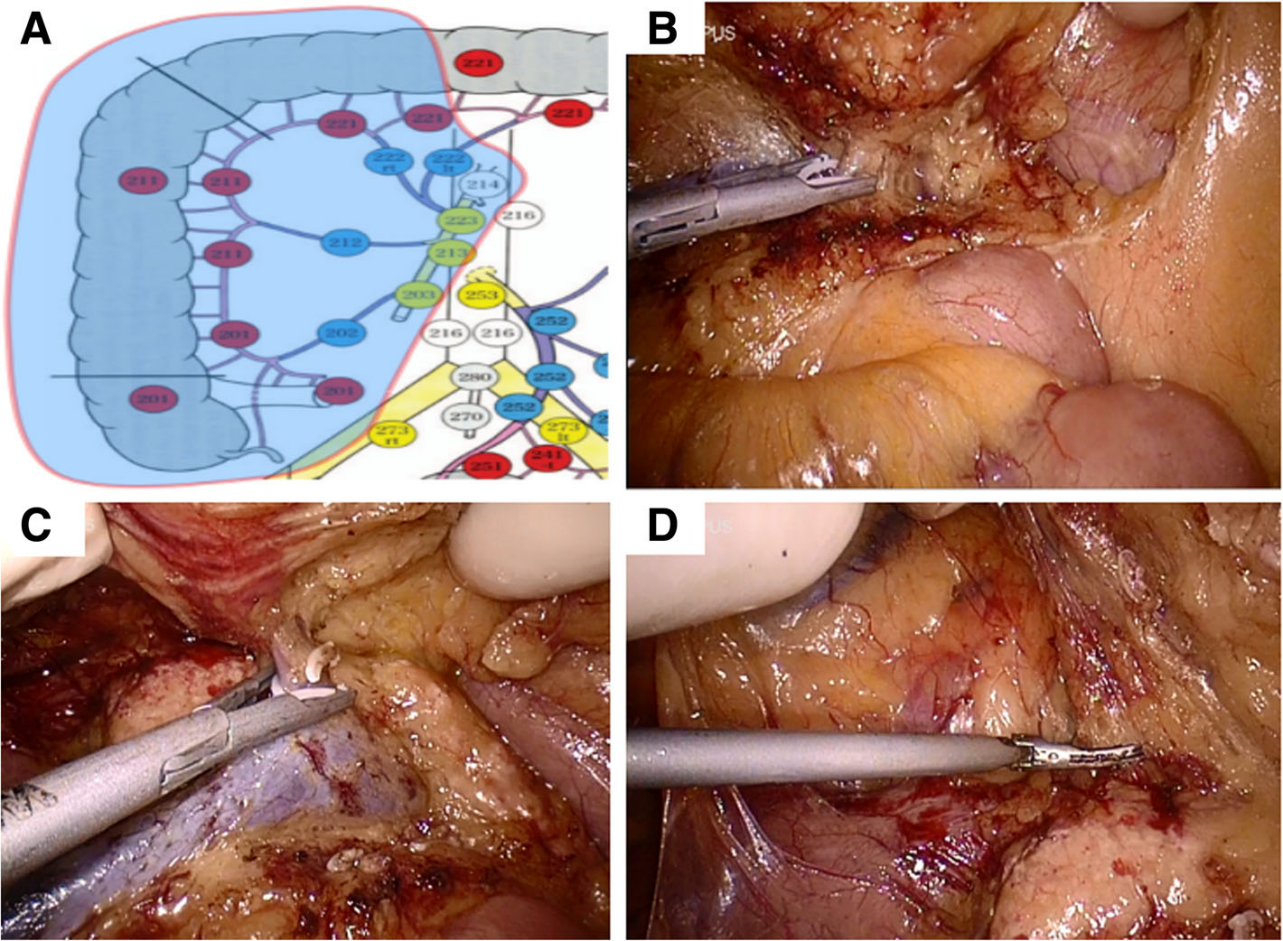
The intracorporeal surgical procedure for the HALS-CME group. **a** The extent of the D3 lymphadenectomy. **b** The neck of pancreas is exposed and middle colic vessels are divided. **c** The Henle’s trunk is identified and handled. **d** The mobilization of the tumor-bearing segment is performed from medial to lateral

In the intracoporeal stage, the principle of European CME and Japanese D3 lymphadenectomy will be followed when right colon resection with en-bloc lymphadenectomy is performed. The extent of the third tier lymph node (N3) dissection (D3) along the SMV is showed in Fig. 4a. The second operation stage under laparoscopy is described as follows:

1. When the transverse mesocolon and the middle colic vessels undergo retracted cephalad retraction, the inferior edge of the neck of pancreas will be identified as a landmark and destination for the following steps.
2. In the first stage, the SMV has been identified and the ileocolic vessels have been divided; dissection is then continued along the left side of the SMV in a repeated unidirectonal/longitudinal manner until the neck of pancreas is exposed and the lesser omental bursa is entered. Meanwhile, the right colic artery and middle colic vessels will also be exposed and divided (Fig. 4b).
3. After exposing the whole length of the neck of pancreas, identifying Henles’ trunk and the pancreaticoduodenal vein, the right colic vein is then divided (Fig. 4c).
4. After completion of the D3 lymphadenectomy along the SMV, the greater omentum will be removed, and mobilization of the right colon mesentery from medial to lateral will be performed with the help of the hand (Fig. 4d).
5. Finally, the specimen will be removed through the hand-assisted port, an end-to-side anastomosis will be fashioned, the mesenteric defect will be closed extracorporeally.

### Control intervention

In this trial, in order to adequately test the novel HALS-CME technique and balance baseline characteristics among groups, two control groups (laparoscopy CME (LAP-CME) and conventional laparoscopy (cLAP)) will be set up.

### The common technique in the LAP-CME and cLAP groups

**Fig. 5:**
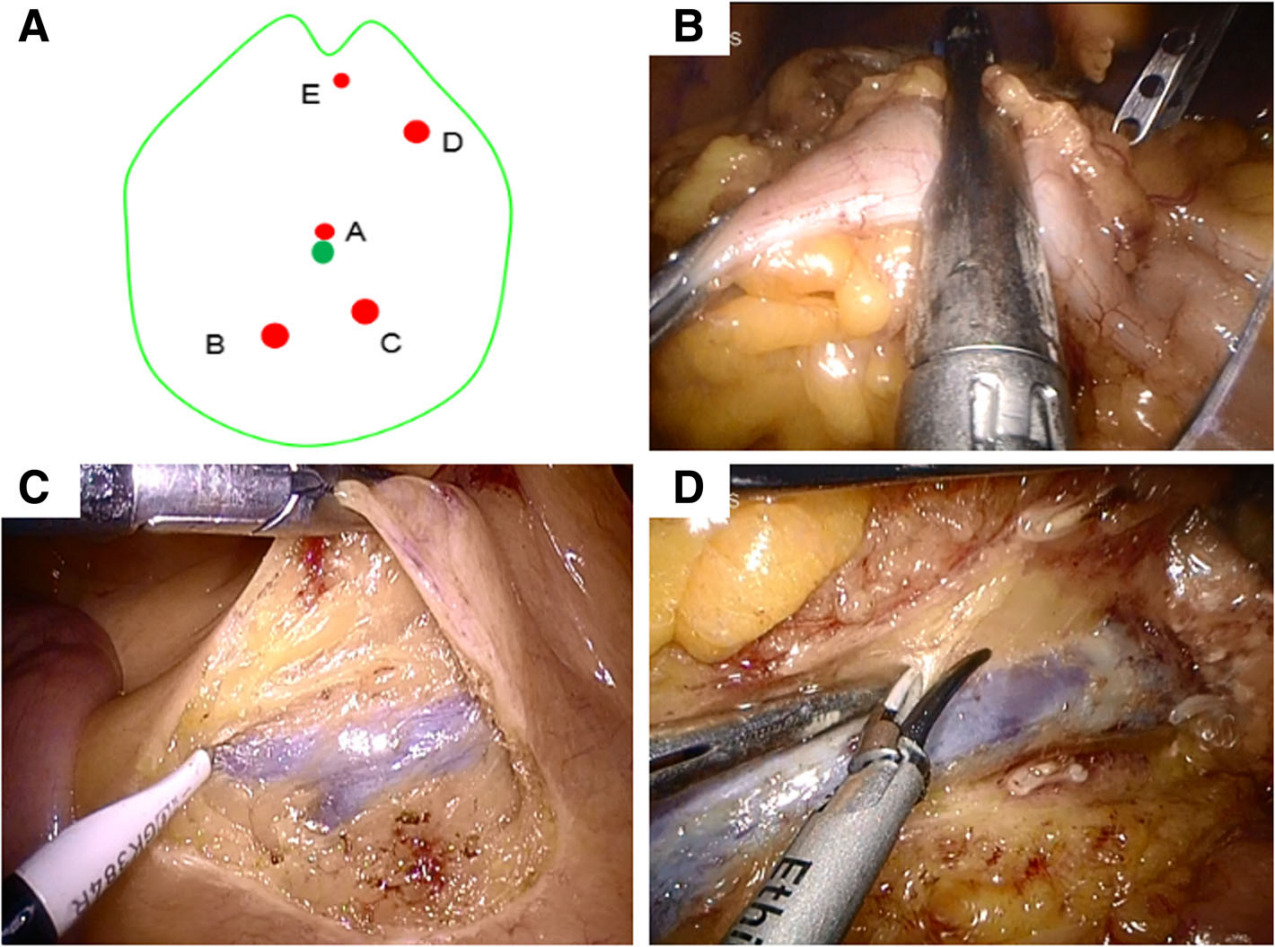
Trocar positions and surgical procedure for the control intervention group. **a** Trocar positions for LAP-CME and cLAP groups. Trocar A = 10 mm, Trocars B–D = 12 mm, Trocar E = 5 mm. **b** The transverse is divided under laparoscopy in the LAP-CME group. **c** The distal ileum is divided under laparoscopy in the cLAP group. **d** The terminal of the SMV is identified under laparoscopy and D3 lymphadenectomy is performed along the SMV in the cLAP group

In the two control groups the position of the patient, the chief surgeon, first assistant, second assistant, and monitor will be the same as described for the experimental group. A five-trocar technique is usually used (Fig. 5a). A camera port (trocar A) will be created through a supraumbilical 10-mm incision and a pneumoperitoneum of 12–15 mmHg will be established through the camera port. Another four transabdominal trocars will also be placed under laparoscopic visualization: two 12-mm trocars (trocars B and C) in the right and left lower quadrant as the surgeon’s dominant operation channel; another 12-mm trocar (trocar D) as the assistant’s dominant operation channel; one 5-mm trocar (trocar E) under the xiphod as the assistant’s nondominant operation channel.

### The different technique in the LAP-CME and cLAP groups

In the LAP-CME group the transverse colon and distal ileum will be firstly divided at the elected sites of resection under laparoscopy, and then the terminal of the SMV will be identified and the ileocolic vessels will be ligated (Fig. 5b). D3 lymphadenectomy along the left side of the SMV in a repeated unidirectonal/longitudinal manner and removing the cancer-bearing segment from the medial to lateral under laparoscopy are similar to the HALS-CME group. The specimen will be dragged out through the midline incision around trocar A and an end-to side anastomosis will be fashioned extracorporeally.

In the cLAP group, after establishing the pneumoperitoneum and exploring the abdomen, the terminal of the SMV will be identified under laparoscopy; the method of D3 lymph node dissection is same as in the HLAS-CME and LAP-CME groups (Fig. 5c and d). After the ileocolic vascular pedicle, right colon vascular pedicle, and middle colon vascular pedicle ligation, the cancer-bearing segment will be removed from the medial to lateral. The involved segment will be dragged out through the midline incision around trocar A. The transverse colon and distal ileum will then be ligated, and an end-to-side anastomosis will be fashioned.

## Blood and peritoneal wash collection

### Blood sample collection

**Fig. 6:**
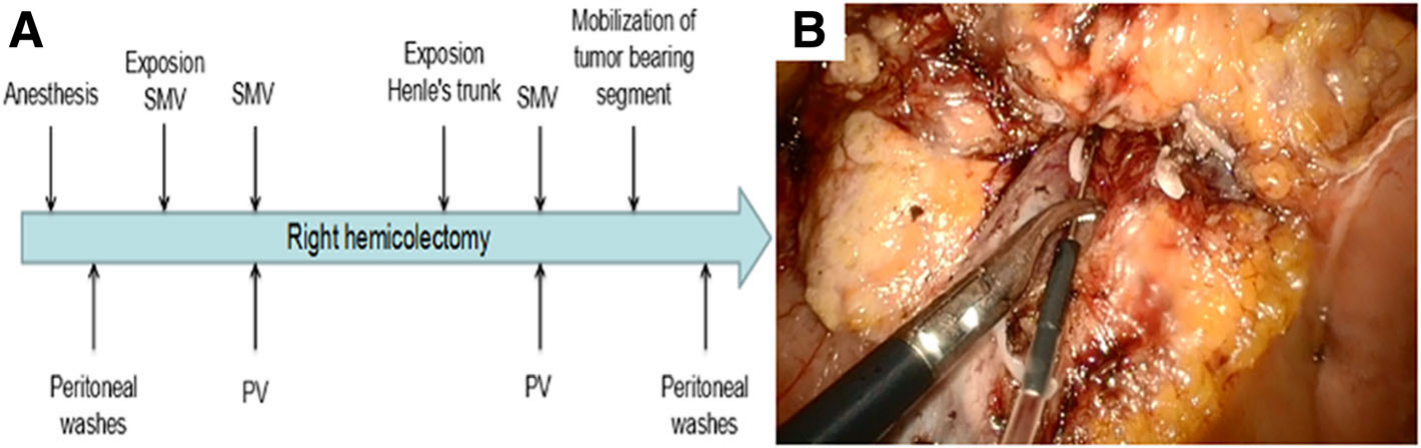
Blood and peritoneal wash collection. **a** Timeframe for collecting blood samples and peritoneal washes. **b** SMV blood is collected using our home-made device under laparoscopy. *PV* peripheral vein, *SMV* superior mesenteric vein

Blood samples from the peripheral blood and SMV will be collected from patients at two different time points (Fig. 6a). After exposing the distal SMV both in the experiment and control groups, blood samples will be extracted simultaneously from the SMV and peripheral vein. The second time point is at after handling the Henles’ trunk and before the mobilization of the cancer-bearing segment. To prevent any contamination of epithelial cells, the initial 2 ml of blood will be discarded from all blood samples and the following 10 ml of blood will be used for RNA extraction. The SMV blood sample will be extracted using our home-made device under larparoscopy (Fig. 6b). Density gradient centrifugation techniques will be used to collect cells.

### Peritoneal wash collection

Peritoneal washes will also be collected at two different time points during the operative period. At the beginning of each operation, 100 ml saline will be introduced into the hepatorenal recess and the Douglas cavity and aspirated after gentle stirring. After complete mobilization of the cancer-bearing segment, another 100-ml peritoneal wash will be aspirated after gentle stirring. Each peritoneal wash will be used for RNA extraction. After the peritoneal washes are centrifuged for 10 min at 1000 × g, cells will be collected and rinsed with phosphate-buffered saline (PBS), dissolved in TRIzol RNA extraction buffer (Invitrogen, Carlsbad, CA, USA), and stored at –80 °C.

### Quantitative TaqMan RT-PCR

**Fig. 7:**
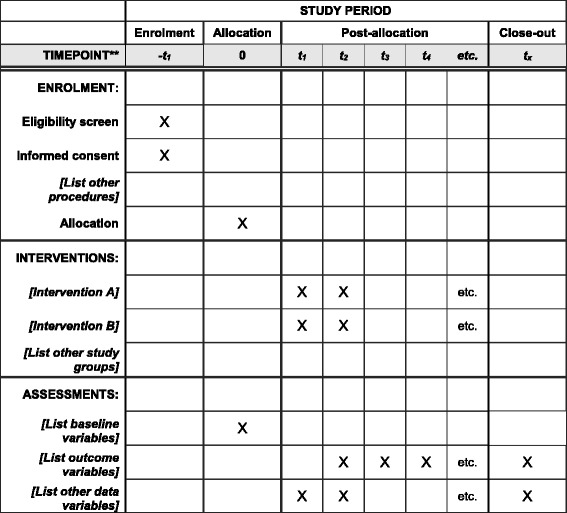
Example template of recommended content for the schedule of enrolment, interventions, and assessments

Total RNA will be extracted through a guanidinium-isothiocyanate-phenol-chloroform-based method. Complementary DNA (cDNA) will be synthesized from total RNA by a PCR instrument Veriti Dx (Applied Biosystems) according to the manufacturer’s instructions. In this reaction, 2 μg total RNA, 4 μl 5× miScript HiSpec Buffer, 1 μl 10× Nucleics Mix, 2 μl miScript Reverse Transcriptase Mix, and no RNA enzyme water will be run in a total reaction volume of 20 μl and incubated at 37 °C for 60 min and 95 °C for 5 min. Two-step RT-PCR with TaqMan probe of CEA, CK20, and Beta-actin will be performed using an Applied Biosystems ViiA7 Real-time PCR System. The probe for CEA and CK20 is ACTB-Taqman, 5’-TGTACGTTGCTATCCAGGCT-3’; primers for CEA, CK20 are as follows: CEA, forward primer, 5’-CAATCTGCCCCAGCATCTTT-3’; reverse primer, 5’-CGGTTGCCATCCACTCTTTC-3’; CK-20, forward primer, 5’-GCAACAGGTCACAGTGAATA3-3’; reverse primer, 5’-CTCAGCTCCGTTAGTTGAAC-3’. The PCR solution (16 μl) will be composed of 1 μl cDNA solution, 2 μl of the forward and reverse primers, 1 μl Taqman probe, 8 μl Taqman Universal Master Mix II (Applied Biosystems), and water to adjust the final volume in each reaction. All reactions will be incubated in a 384-well plate at 95 °C for 6 min, followed by 45 cycles of 95 °C for 10 s, 60 °C for 10 s, and 72 °C for 30 s. All samples will be performed in triplicate. The actin gene will be used as a control to normalize differences in total RNA levels in each sample. The relative amount of each mRNA to actin RNA will be expressed using the equation 2^–ΔΔ^CT, where ΔΔCT = (ΔCt mRNA – ΔCt actin). E﻿xample template of recommended content for the schedule of enrolment, interventions, and assessments is shown in Fig. 7.

## Discussion

Since total mesorectal excision (TME) has been accepted worldwide, the survival from rectal cancer has significantly improved, and is even better than for colon cancer in some countries [14–17]. In 2007, CME with CVL for colon cancer was suggested in Western countries which follows similar oncological principles as TME does for rectal cancer [6]. The principles of CME require resection of the affected colon with its associated lymphovascular supply through complete removal of the mesocolon as an envelope to minimize the risk of spillage of tumor cells into the peritoneal cavity and central ligation of the vascular supply at its origins to increase lymph node harvest. In a series of studies, CME has shown an improved survival and reduced local recurrence when compared with standard surgery [7, 8, 18, 19].

Realizing that removing draining lymph nodes potentially eliminates the probability of leaving behind residual disease, which has implications for local control and survival, more emphasis is placed on lymphadenectomy in Eastern countries. Moreover, a D3 extended lymphadenectomy is considered the standard of care for clinical stage II and III colon disease in Asian countries, especially in Japan [20]. Actually, both CME and D3 lymphadenectomy follow the same oncological principles, mainly including excision of the mesocolon within an intact fascial envelope and central vascular ligation. Furthermore, both techniques have shown impressive outcomes as compared with standard excision.

For left-sided colon cancer, exposure and ligation the origin of the inferior mesenteric artery is not difficult, as the TME principles are commonly followed. However, in order to identify the origins of the vessels supplying the right-side colon and perform true D3 lymphadenectomy, the head of the pancreas, the anterior surface of the SMV, and the superior mesenteric artery (SMA) should be fully exposed, which are all technical challenges and more traumatic, leading to longer operation times than the standard operation [21, 22]. The original CME initially described by Hohenberger was performed via a laparotomy. Due to laparoscopic surgery with short- and long-term benefits, there are a series of studies looking at the feasibility of laparoscopic-CME especially for right colon cancer. Finally, although these studies have shown the feasibility and safety of this procedure with acceptable morbidity and oncological outcomes, laparoscopic right hemicolectomy with CME and true center vascular ligation for right colon cancer remains technically challenging especially for obese patients [9, 22, 23].

Since HALS returns the sense of touch to the surgeon, this procedure may represent a valid alternative approach to standard laparoscopy. Furthermore, HALS has been shown to eliminate a substantial part of the technical challenges of standard laparoscopy along with having an acceptable learning curve and reducing operative time, but patient morbidity rates and recovery are comparable with standard laparoscopy [12, 13]. However, this HALS technique allows for hand assistance during laparoscopic surgery and tactile sensation of the lesion, and people argue that this approach is against the “no-touch” isolation technique.

The virtue of the “no-touch” isolation technique is another controversial issue in the field of colorectal disease. In 1952, Barnes first described and adopted a special technique for resection of right colon cancer: ligation of the vascular pedicles and division of the bowel before handling the cancer-bearing segment [24]. This special technique was first named a “no-touch” isolation technique by Turnbull in 1953, based on previous clinical and basic research [25]. The aim of this technique is to reduce cancer cells flowing from the primary tumor site to the liver and other organs by ligation of the vascular pedicles first. However, since then, the value of the “no-touch” technique in colon surgery has always been debated. In a retrospective analysis, although Turnbull et al. had demonstrated that “no-touch” isolation resection could greatly improve survival rates compared with conventional manipulative resection, these results might be due to more extended resections in the no-touch group [25]. The only prospective randomized controlled trial did suggest a limited benefit of the “no-touch” isolation technique with regard to the overall survival, and a tendency for reduction in occurrences of liver metastases [26]. Another randomized controlled trial is currently underway in Japan to demonstrate the superiority of the special technique (JCOG1006) [27]. Whether surgical manipulation of the cancer-bear segment would increase the detachment and circulation of tumor cells into the peripheral circulation is also still debated. Hayashi et al. [28] demonstrated that the “no-touch” isolation technique may prevent cancer cells from being shed into the portal circulation by using mutant-allele-specific amplification, while Garcia-Olmo et al. [29] found CEA products only in one of eighteen patients who underwent conventional surgery by using RT-PCR.

In this trial, the novel HALS-CME procedure, which takes full advantages of the HALS technique and potential “no-touch” isolation technique, has several technical merits:

1. After transecting the distal ileum and its mesentery, the distal end of the superior mesenteric vessels are easily exposed, especially for obese patients.
2. Transecting the transverse colon and returning the two ends back into peritoneal cavity, the neck of the pancreas can be easily observed and be a landmark during lymph node dissection along the left and surface of the SMV, which is true D3 lymphadenectomy.
3. It is well known that there are fewer venous branch drains into the left of the SMV; dissection along the left axis of the SMV in a repeated unidirectonal/longitudinal manner facilitates location and ligation of the root of the ileocolic vessels, right colic vessels, and Henle’s trunk, avoiding injuring these branches.
4. After vascular pedicles, the transverse colon, and the distal ileum are ligated, the cancer-bearing segment is manipulated from medial to lateral, which is more consistent with the “no-touch” isolation technique described by Barnes [24]. The total “no-touch” isolation technique appears to have potential benefits for decreasing tumor cells spreading into the portal vein and circulatory system during the operative manipulation.
5. After transecting the bowel and ligating feeding vessels, the medial to lateral approach is adopted to avoid direct contact with the tumor. The surgeon’s hand provides better retraction for mobilization of the involved colon and dissection along the Toldt’s fascia, which shortens the operation time.

There are potential limitations in this trial. 1) The trial will be carried out in only one center, which may impact participant recruitment and limit the applicability of the outcomes to other centers. 2) The open-label nature of the trial may cause biased estimates of the treatment effect. However, it is not possible to blind the surgeon for the special nature of nonpharmacologic trials. Although the surgeons, researchers, and participants are not blinded, the pathologists, statisticians, and follow-up staff will be blinded to the group assignment, which contributes to reducing the treatment effect estimation bias. 3) The relatively short follow-up time has an impact on comparing long-term outcomes. In summary, if the feasibility, short-term safety, long-term oncological safety, and potential total “no-touch” isolation technique benefits of HALS-CME are verified, this technique could be recommended as a new approach to overcome the technical challenges in right hemicolectomy for right colon cancer.

## Trial status

This trial was initiated in December 2015 and is currently recruiting patients.

## Additional file

Additional file 1: SPIRIT 2013 Checklist: Recommended items to address in a clinical trial protocol and related documents. (DOC 116 kb)

## Abbreviations

AJCC: American Joint Committee on Cancer
ASA: American Society of Anesthesiologists
CA19-9: Carbohydrate antigen 19-9
cDNA: Complementary DNA
CEA: Carcinoembryonic antigen
cLAP: Conventional laparoscopic surgery with CME and D3 lymphadenectomy
CME: Complete mesocolic excision
CONSORT: Consolidated Standards of Reporting Trials
CRF: Case report form
CRP: C-reactive protein
CT: Computed tomography
CVL: Central vascular ligation
DFS: Disease-free survival
ECOG: Eastern Cooperative Oncology Group
HALS: Hand-assisted laparoscopic surgery
HALS-CME: Hand-assisted laparoscopic surgery with complete mesocolic excision, D3 lymphadenectomy, and total “no-touch” isolation
IL: Interleukin
LAP-CME: Laparoscopic surgery with CME, D3 lymphadenectomy, and total “no-touch” isolation
NCCN: National Comprehensive Cancer Network
OS: Overall survival
PBS: Phosphate-buffered saline
PCT: Procalcitonin
PFS: Progression-free survival
RT-PCR: Real-time polymerase chain reaction
SMA: Superior mesenteric artery
SMV: Superior mesenteric vein
TEM: Total mesorectal excision
